# Practical Considerations and Challenges Involved in Surfactant Enhanced Bioremediation of Oil

**DOI:** 10.1155/2013/328608

**Published:** 2013-11-24

**Authors:** Sagarika Mohanty, Jublee Jasmine, Suparna Mukherji

**Affiliations:** Centre for Environmental Science and Engineering, Indian Institute of Technology Bombay, Powai, Mumbai 400 076, India

## Abstract

Surfactant enhanced bioremediation (SEB) of oil is an approach adopted to overcome the bioavailability constraints encountered in biotransformation of nonaqueous phase liquid (NAPL) pollutants. Fuel oils contain *n*-alkanes and other aliphatic hydrocarbons, monoaromatics, and polynuclear aromatic hydrocarbons (PAHs). Although hydrocarbon degrading cultures are abundant in nature, complete biodegradation of oil is rarely achieved even under favorable environmental conditions due to the structural complexity of oil and culture specificities. Moreover, the interaction among cultures in a consortium, substrate interaction effects during the degradation and ability of specific cultures to alter the bioavailability of oil invariably affect the process. Although SEB has the potential to increase the degradation rate of oil and its constituents, there are numerous challenges in the successful application of this technology. Success is dependent on the choice of appropriate surfactant type and dose since the surfactant-hydrocarbon-microorganism interaction may be unique to each scenario. Surfactants not only enhance the uptake of constituents through micellar solubilization and emulsification but can also alter microbial cell surface characteristics. Moreover, hydrocarbons partitioned in micelles may not be readily bioavailable depending on the microorganism-surfactant interactions. Surfactant toxicity and inherent biodegradability of surfactants may pose additional challenges as discussed in this review.

## 1. Introduction

Widespread use of petroleum hydrocarbons, generation of petroleum wastes in large quantities, and their recalcitrance lead to the accumulation of these pollutants [1, 2]. Persistence of petroleum hydrocarbons in the aqueous environment eventually affects the flora and fauna of the affected habitat, while subsurface soil contamination eventually leads to contamination of groundwater. Several treatment options such as incineration, solvent extraction, and pump and treat options have been used for the remediation of oil contaminated soil; however, bioremediation is typically more cost effective compared to these physicochemical options. Although structurally complex, several of the petroleum hydrocarbon constituents in oil can be completely mineralized or transformed through microbial activity [3, 4]. Thus, the recalcitrance of petroleum hydrocarbons is primarily due to the inaccessibility of these compounds to most microorganisms. In such a scenario, surfactants may be added to alter the properties of solution interfaces, thereby enabling the access of hydrocarbons to the microorganisms [5–7]. This is referred as “Surfactant Aided Bioremediation” or “Surfactant Enhanced Bioremediation” (SEB). 

Surfactants are amphiphilic in nature. They lower the interfacial tension at the oil-water interface and the surface tension of water and thus favor mass transport of hydrocarbons from the oil phase into the aqueous phase. Primarily, three mechanisms are responsible for enhancing bioavailability by affecting the distribution of hydrocarbons [5, 8], that is, emulsification, pseudo solubilization, and facilitated transport. Reduction in interfacial tension between the aqueous phase and oil results in emulsification. As a result, the interfacial area between the phases is increased and this facilitates mass transport of the hydrocarbons from the oil phase to the aqueous phase. Solubilization refers to the increase in solubility of the hydrocarbons due to the partitioning within surfactant micelles. Micelles consist of a circular arrangement of surfactant monomers such that the hydrophobic tails of the monomers are oriented towards the center. Hydrocarbons from the oil phase are thus preferentially partitioned in the hydrophobic core of the micelles. Facilitated transport refers to all other types of processes which favor mass transfer of hydrocarbons either by the interaction of oil with a single surfactant monomer or surfactant aggregates or by the interaction of surfactants with sorbed oil. All these mechanisms are very closely linked, and, at times, it is very difficult to distinguish between them. 

More recently, it has been highlighted that surfactants not only affect the distribution of oil but also affect the microbial cell surface properties, and such interactions may have both beneficial and detrimental effects on the biodegradation of hydrocarbons [9–13]. It is also important to select microorganisms that are resistant to the surfactant, that is, those that are not adversely affected by its toxicity. Some hydrocarbon degrading microorganisms are also reported to degrade the surfactants; however, preferential degradation of surfactants may hinder the uptake of hydrocarbons [13, 14]. Biodegradability of surfactants subsequent to oil degradation is a desirable trait that can eventually lead to the sustainable application of this technology. However, accumulation of toxic intermediates during surfactant degradation may pose additional challenges. Partial degradation of alkylphenol ethoxylates (APEOs) is reported to cause the accumulation of intermediates such as nonylphenol (NP) and octylphenol (OP) that are known to cause endocrine disruption [15, 16]. Although SEB has been successfully applied for the biological treatment of oily waste using microbial consortia [17, 18], there are still numerous challenges that restrict its usage in oil spill bioremediation scenarios. Controlled lab scale studies have revealed various important interactions and effects that may adversely affect oil remediation in the presence of surfactants. This review highlights the various challenges that need to be considered for the successful application of SEB. The primary focus is on the application of chemical surfactants for SEB. 

## 2. Microbial Degradation of Petroleum Hydrocarbons

Microorganisms degrading complex petroleum mixtures, such as diesel, crude oil, gasoline, heavy oil, and lubricating oil, are abundant in nature. However, complete mineralization of such complex mixtures is normally not attained. Some microorganisms may degrade aliphatic hydrocarbons and some may only degrade aromatic hydrocarbons, while some other microorganisms may have the capability to degrade both aliphatics and aromatics [19–21]. Commonly, a microbial consortium consisting of microorganisms which degrade different types of substrates is used for better degradation of complex mixtures. Although a microbial consortium is commonly employed in field scenarios, pure culture studies can reveal much information on the interactions that occur during oil uptake. The major limitations to microbial degradation of petroleum hydrocarbons include culture specificities and consortia effects, multiple substrate interaction effects, and bioavailability limitations [22].

### 2.1. Culture Specificities and Consortia Effects

Since hydrocarbons constitute a ubiquitous class of natural compounds, microorganisms degrading them, such as bacteria, fungi and algae, are widely distributed in nature. Rosenberg [23] estimated that a typical soil or ocean sediment has 10^4^–10^6^ hydrocarbon degrading microorganisms per gram. Hence, the remediation of oil contaminated area can be done by the enrichment of local microorganisms without any external seeding or use of genetically engineered microorganisms. However, complete mineralization is possible only when the microorganisms specific for utilizing the different components of oil are present in the contaminated site. Alkanes are degraded by most microorganisms. Cycloalkanes and aromatics, such as polynuclear aromatic hydrocarbons (PAHs), are degraded only by specific microorganisms. Such specificities of the microorganisms and the presence of the contaminants at concentrations toxic to some microorganisms may hinder the degradation process. Further, the interrelationship of the microorganisms involved also affects the degradation process. During the degradation of crude oil, Van Hamme and Ward [17] observed that physical and metabolic interactions between a coculture of *Rhodococcus* sp. strain F9-D79 and *Pseudomonas* sp. strain JA5-B45 enhanced the degradation of crude oil only slightly over that achieved by the individual cultures. Thus, culture specificities and interrelationship amongst the cultures of a consortium determine the fate of oil degradation in a contaminated site.

### 2.2. Multiple Substrate Interaction Effects

Microbial degradation of petroleum hydrocarbons may also be affected by interaction effects of the multiple substrates present in complex hydrocarbon mixtures [24]. This has been observed not only for mixtures of recalcitrant toxic chemicals (as encountered in bioremediation) but also for mixtures of readily degraded pollutants (wastewater treatment) and mixtures of sugars (fermentation). Mixture effects can be understood by considering the metabolic role of each compound for the microorganisms involved. The substrate interaction patterns include no interaction, increased growth at low substrate concentration [25], induction of degradative enzymes, competitive inhibition, toxicity, and the formation of toxic intermediates by nonspecific enzymes [26–28].

Several models have been proposed to predict the type of interaction in such complex substrate systems. Most models have been tested with only two substrates, and their applicability to larger mixtures has been assumed without validation. However, a few models have been proposed and tested for multicomponent mixtures with more than 2 components. Some examples are growth of a mixed culture on benzene, toluene, ethylbenzene, and *o*- and *p*-xylene (BTEX compounds) [29] and the biodegradation of three polynuclear aromatic hydrocarbons (PAHs) [30]. Guha et al. [30] proposed a multisubstrate Monod kinetic model for determining substrate interactions between PAHs. The main assumption of their model was that the multisubstrate system is comparable to the single-substrate system with respect to physiological state. This assumption may be valid for the mixed culture system, provided that the substrates are utilized by a common enzyme system. Reardon et al. [31] found that, for pure cultures growing on aromatic chemical mixtures, neither a no interaction nor a competitive inhibition model accurately predicts the mixture kinetics. To overcome this difficulty, they developed a model which used model parameters from single- and dual substrate mixture experiments to predict the outcome of the 3-substrate mixture experiment. They also found that the interactions between species had a significant impact on the biodegradation kinetics, and that the nature of these interactions was dependent on the growth substrates. 

### 2.3. Bioavailability Issues

An important factor affecting oil biodegradation is the accessibility of the petroleum hydrocarbons to the microorganisms. Bioavailability is of concern due to the aqueous and nonaqueous biphasic nature of the system. The utilization of hydrocarbons by microorganisms can take place either through direct interfacial uptake by the attachment of microbial cells to the nonaqueous phase liquid- (NAPL-) water interface or after mass transfer of the NAPL to the aqueous phase [32]. Biodegradation rates during uptake from the aqueous phase are dependent on phase equilibrium and mass transfer. Mass transfer limits the biodegradation rates if the mass transfer rates are slow since both processes occur in succession. Biodegradation rates can be enhanced by the secretion of biosurfactants by the microorganisms themselves. Thus, biosurfactants play a significant role in enhancing the bioavailability of petroleum hydrocarbons. Biosurfactants act by lowering the interfacial and surface tensions, and they also play a significant role in stabilizing oil-in-water emulsion [33–35]. Direct interfacial uptake has been reported for the uptake of n-alkanes, fuel oil, and solid PAHs [32, 36]. Such uptake is facilitated by cultures that possess or can induce high cell surface hydrophobicity and cultures that show enhanced adherence to n-hexadecane in bacterial adhesion to hydrocarbon (BATH) assay. 

## 3. Surfactant Aided Biodegradation of Petroleum Hydrocarbons

**Figure 1:**
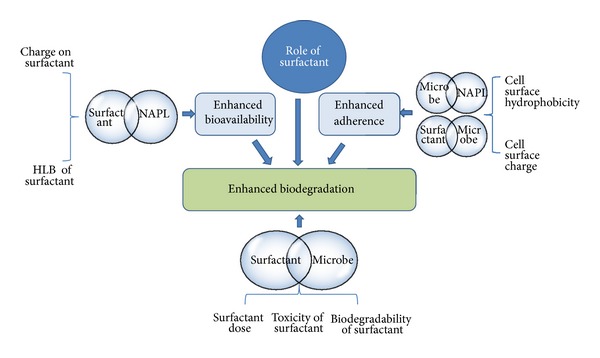
Schematic diagram illustrating the complex interplay of interactions between surfactant, microorganism, and substrate during SEB.

Surfactant aided biodegradation is affected by a complex interplay of factors as illustrated in Figure 1. These factors play a significant role in controlling the process, and subsequent successful implementation of surfactant aided biodegradation in contaminated sites. The important factors include: selection of appropriate surfactant, selection of appropriate microbial culture, toxicity of the surfactant, and biodegradability of the surfactant.

### 3.1. Surfactant Selection: SEB through Emulsification and Micellar Solubilization

It is essential that an appropriate surfactant is selected for remediation of a particular type of petroleum waste in any scenario. The surfactant structure, its hydrophile-lipophile balance (HLB) number, dose, and its mechanism of action, that is, emulsification versus micellar solubilization, may affect the outcome of SEB. Surfactants are mainly classified into four types depending on the charge on the hydrophilic head group, that is, cationic, anionic, nonionic, and zwitterionic [7]. In synthetic surfactants, the hydrophobic portion of the surfactant may be comprised of paraffins, olefins, alkylbenzenes, alkylphenols, and alcohols. In cationic surfactants, the hydrophilic head group such as the quaternary ammonium group carries positive charge. The carboxylic group or sulphonate group imparts a net negative charge to anionic surfactants. In nonionic surfactants, the hydrophilic head groups mainly consist of sucrose, polyoxyethylene, or polypeptide. Zwitterionic surfactants are those which have both cationic and anionic groups and these surfactants mainly consist of one or more hydrophilic head or hydrophobic tail. Zwitterionic surfactants have good potential for enhancing the solubilization capacity at low doses [7].

In addition to synthetic surfactants, biosurfactants secreted by microorganisms may also be utilized for bioremediation of oil and petroleum hydrocarbons. Several microbial species are known to produce biosurfactants, and the chemical structure of these biosurfactants is reported to vary widely [6]. These include trehalose lipids produced by *Mycobacterium *sps. and *Rhodococcus erythropolis*, rhamnolipids produced by *Pseudomonas *sps., Sophorolipids produced by *Candida apicola*; lipopolysaccharides produced by *Acinetobacter calcoaceticus* (RAG1), and phospholipids produced by *Thiobacillus thiooxidans.* Biosurfactants are mostly anionic or nonionic. Chemically, the hydrophilic head group of biosurfactants may consist of a carbohydrate, peptide, amino acid, phosphate, carboxylic acid, or alcohol while the hydrophobic tail may consist of fatty acids, hydroxy fatty acids, or *α*-alkyl-*β*-hydroxy fatty acids. For total petroleum hydrocarbons (TPHs) associated with soil, biosurfactants such as rhamnolipids and surfactin have been found to remove TPH at higher rates compared to the synthetic surfactants [37]. Biosurfactants are preferable compared to synthetic surfactants as they are easily biodegradable. However, the implementation of SEB using biosurfactants becomes difficult due to their high cost of production and extraction. 

**Figure 2:**
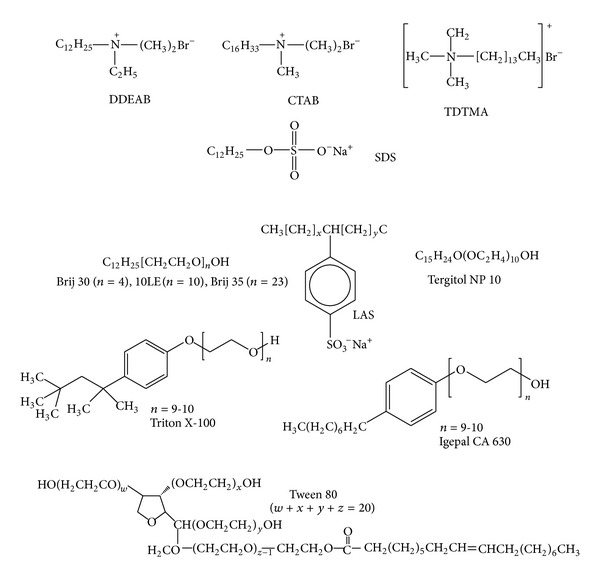
Structure of chemical surfactants commonly used in bioremediation.

**Figure 3:**
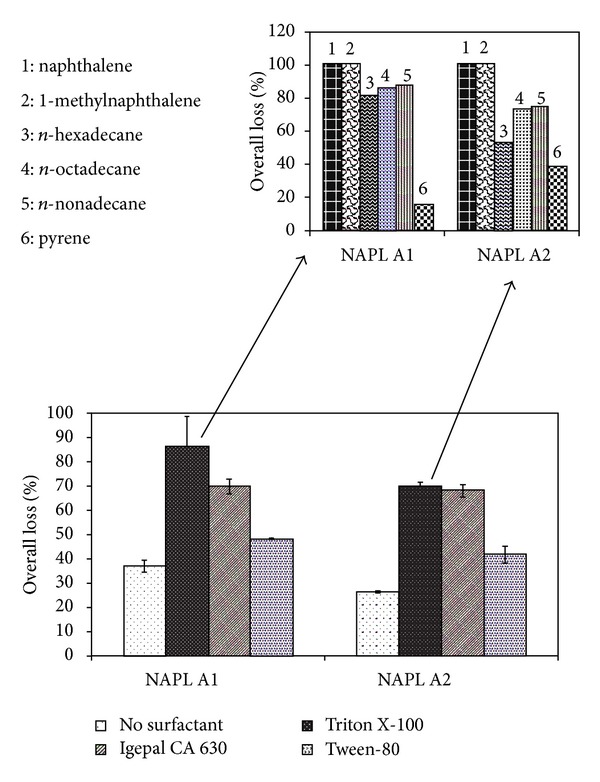
Degradation of NAPLs by naphthalene degrader *Burkholderia multivorans*-NG1 in the absence and presence of surfactants and component-wise degradation in presence of Triton X-100. Prepared based on data published in Mohanty and Mukherji [11, 12].

Table 1 summarizes studies on the application of various surfactants for the degradation of petroleum hydrocarbons, polynuclear aromatic hydrocarbons (PAHs), oil, and model nonaqueous phase liquids composed of hydrocarbons. The structure of some of the chemical surfactants is illustrated in Figure 2. Nonionic surfactants have been studied more extensively. Triton X-100 and Igepal CA-630 are structurally similar nonionic surfactants (octylphenoxy polyoxyethylene ethanol) that have been widely applied. Other nonionic surfactants commonly applied in bioremediation include Tween 80, a polyoxyethylene sorbitan monooleate with 20 EO units, and Tergitol NP-10, a nonylphenol ethoxylate with 10 EO groups in the ether side chain. Nonionic surfactants are commonly characterized in terms of the HLB (hydrophile lipophile balance) number, which is a measure of the hydrophilicity of the surfactants measured on a scale of 0 to 20, where HLB of 20 is assigned to the most hydrophilic surfactant. Increasing number of EO units imparts greater hydrophilicity and increases the HLB number. Typically, surfactants with HLB between 7, and 11 are reported to form water in oil emulsion whereas those with HLB number between 12 and 16 are reported to form oil in water emulsion [38]. Triton X-100, Igepal CA-630, Tween 80, and Tergitol NP-10 are reported to have HLB of 13.5, 13, 15, and 14, respectively. However, emulsification is also affected by the type of oil and the concentration of surfactant used. Surfactants with higher HLB number primarily act through micellar solubilization. The extent of solubilization is dependent on the surfactant concentration over and above the critical micelle concentration (CMC). CMC refers to the concentration of a surfactant at which the surface tension reduces to minimum and the surfactant micelle formation is initiated. In a study on the degradation of model NAPLs composed of aliphatic and aromatic hydrocarbons, Mohanty and Mukherji [11, 12] reported the emulsification of the NAPLs by Triton X-100 and Igepal CA-630 which was associated with enhancement in NAPL degradation by the naphthalene degrader, *Burkholderia multivorans* (NG1) as illustrated in Figure 3. *Burkholderia multivorans* (NG1) depicted negligible degradation of aliphatic hydrocarbons in the absence of surfactants. In the presence of the emulsifying surfactants, Triton X-100 and Igepal CA-630, significant degradation of aliphatic hydrocarbons was observed. In contrast, in the presence of Tween 80, the *Burkholderia *sp.illustrated much lower degradation since Tween 80, characterized by a higher HLB value, did not cause emulsification. The NAPLs differed from each other in the naphthalene: n-hexadecane ratio such that for NAPL A1 it was 1 : 1 and for NAPL A2 it was 1 : 3. Song and Bielefeldt [39] also recommended the use of nonionic surfactants with midrange HLB values for surfactant enhanced aquifer remediation and reported inhibitory effects of surfactants with either very low or very high HLB values. In contrast, Torres et al. [40] demonstrated the beneficial effect of low HLB value nonionic surfactants on bioremediation of diesel from aged soils in microcosm experiments by naturally occurring soil bacteria, where the beneficial effect was attributed to the formation of water in oil emulsions. 

Surfactant type not only affects the mechanism through which bioavailability is enhanced but also affects the structure of the micelles and the solubilization of components within micelles [41]. The effectiveness of micellar solubilization of hydrocarbons is depicted in terms of the molar solubilization ratio (ratio of solubility enhancement above CMC to that of surfactant concentration above CMC) and the micelle water partition coefficient (mole fraction in micelles to mole fraction in the aqueous phase at equilibrium). Although the solubilization of hydrocarbons primarily happens in the micellar core, PAHs with resonating *π* electrons can also form weak bonds with oxygen containing head groups in the shell region of nonionic surfactant micelles. In cationic surfactants, PAHs may form bonds with the cationic head groups and may thus exist both at the micelle water interface and in the micellar core. Micellar solubilization of naphthalene and pyrene was found to be affected by the charge on the hydrophilic group, the hydrophobic chain length, and the geometry of micelles. For comparable hydrophobic chain lengths, nonionic surfactants typically showed higher solubilization. However, pyrene solubilization in ionic surfactants, such as sodium dodecyl sulphate (SDS, anionic), cetyltrimethyl ammonium bromide (CTAB, cationic) and dodecylethyldimethyl ammonium bromide (DDEAB, cationic), was found to be enhanced in the presence of naphthalene [41]. Such change in micelle water partition coefficients in multi-solute systems is also likely to impact SEB. 

However, enhancement in solubilization in the presence of surfactants does not always lead to enhanced biodegradation. Some surfactants trap oil/hydrocarbons in the hydrophobic core of the micelles and make them unavailable to the microorganism [30, 42]. The rate of mass transfer from the micelles to the aqueous phase [12] and the location of the hydrocarbons in the micelles (core versus shell) are likely to affect the rate of biodegradation. In experiments with phenanthrene sorption on a *Burkholderia *sp., Lanzon and Brown [43] illustrated how the sorption of a Brij 30 surfactant on bacteria in the form of hemimicelles enhanced phenanthrene sorption on bacteria. Such sorption in the form of hemimicelles rather than monomers is expected to enhance the bioavailability of the hydrocarbons. They suggested that the fraction of micellar HOC that is bioavailable is directly related to the formation of hemimicelles on bacterial cell surfaces. In some cases, bioavailability enhancement through micellar solubilization and emulsification may also cause toxicity to the microorganisms and hinder the biodegradation of petroleum hydrocarbons. Such an effect was observed during the treatment of emulsified diesel in a rotating biological contactor in the presence of Triton X-100 by *Burkholderia multivorans* [44], although Triton X-100 was nontoxic to the culture. Similar toxic effect has been reported due to the solubility enhancement of PAHs in surfactant micelles [45]. 

Surfactant dose impacts the mode of action of surfactants and affects biodegradation. At high dose, surfactants may adversely affect the microorganisms responsible for the degradation of petroleum hydrocarbons and oil due to their inherent toxicity. Moreover, higher dose also translates to higher cost. The administration of appropriate surfactant dose is essential for effective bioremediation. Surfactants with lower CMC value are preferred for bioremediation since solubilization is effective only for surfactant concentration above the CMC. Surfactant concentration beyond the CMC is linearly related to increase in degradation up to a concentration that is toxic to the microorganism. Enhanced degradation with increasing surfactant dose above CMC due to micellar solubilization was demonstrated during the degradation of phenanthrene and pyrene using nonionic and anionic surfactants, Tween 20, Tween 80, Triton X-100, and SDS [46]. Tween 80 showed higher solubilizing capability than Triton X-100 and Tween 20, and Tween series surfactants showed greater biodegradation. Li and Chen[47] reported solubilization enhancement and degradation of phenanthrene by a marine bacteria in presence of nonionic surfactants Tergitol 15-SX (X = 7, 9, and 12). However, for the same initial phenanthrene concentration, an increase in surfactant concentration decreased the biodegradability due to the low bioavailability of phenanthrene from surfactant micelles. Bautista et al. [48] illustrated significant enhancement in the degradation of low MW two and three ring PAHs in the presence of 1% surfactant by *Enterobacter *sp.*, Pseudomonas *sp., and *Stenotrophomonas *sp., where the nonionic surfactants used were Tween 80, Triton X-100, and Tergitol NP-10. The increased degradation rates were attributed to micellar solubilization. 

**Figure 4:**
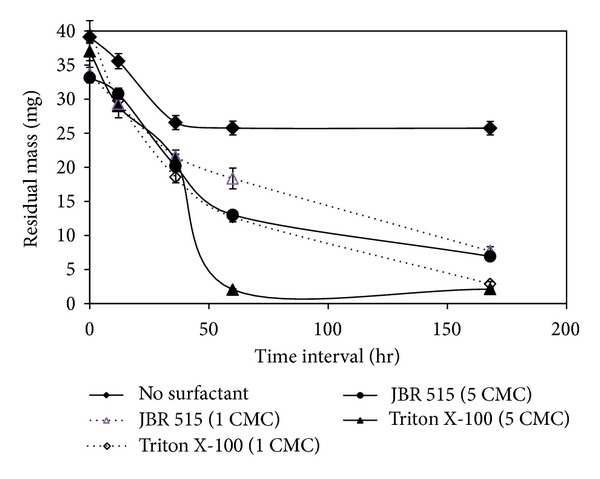
Impact of surfactant dose on degradation of NAPL A1 by *Burkholderia multivorans* (NG1). Prepared based on data published in Mohanty and Mukherji [12].

Irrespective of the mechanism of action, increase in surfactant dose was found to increase the degradation rate of model NAPL A1 (0.1%) by *Burkholderia multivorans* (NG1) as illustrated in Figure 4 [12, 44]. Triton X-100 was reported to act via emulsification, whereas the rhamnolipid biosurfactant JBR-515 acted through micellar solubilization. The increase in surfactant dose induced rapid rate of mass transfer which increased the rate of biodegradation by the microorganism. Similar observations were reported by Mohanty and Mukherji [11] for *Burkholderia cepacia* (ES1) in the presence of Triton X-100. They reported that the rate of degradation of a model NAPL (NAPL A2) was faster as the surfactant concentration was increased from 0 to 5 CMC. 

While micellar solubilization is the predominant phenomenon at the surfactant concentration above CMC, low surfactant concentration may also impact SEB by affecting the microorganism-substrate interaction. The role of surfactant type on such interactions was investigated by Rodrigues et al. [62] using Tween 20 (nonionic), Tergitol NP-10 (nonionic), SDS (anionic), and CTAB (cationic) during the degradation of fluoranthene and anthracene using *Pseudomonas putida* ATCC strain 17514. In general, low surfactant concentrations stimulated the degradation of PAHs even for surfactants such as Tween 20 that could be degraded by the culture. However, SDS had different impacts on fluoranthene and anthracene degradation such that it decreased fluoranthene degradation but increased anthracene degradation. CTAB had a detrimental effect on the degradation of both of the PAHs and also hindered culture growth. 

In contrast, Jin et al. [73] reported the inhibition of phenanthrene biodegradation by *Mycobacterium spp.* KR2 in the presence of low surfactant concentration less than 40 mg/L. The nonionic surfactants used were Tween 80, polyoxyethylene (POE, 4) sorbitan monooleate (Brij 30), POE (10) sorbitan monooleate (10LE), and POE (23) sorbitan monooleate (Brij 35). The anionic and cationic surfactants, linear alkyl benzene sulphonate (LAS), and tetradecyl trimethyl ammonium bromide (TDTMA) were also used. The presence of surfactants decreased the mineralization of phenanthrene and caused the release of phenanthrene intermediates in the form of volatile organic compounds (VOCs), although, culture growth was slightly enhanced due to preferential utilization of the surfactants as growth substrate when present at low concentration. 

In subsurface contamination scenarios, surfactant sorption also needs to be considered. Surfactants that depict lower sorption would be more effective in enhancing the mobility of the oil trapped in soil pores and in enhancing the bioavailability of oil. Anionic and nonionic surfactants typically depict lower sorption to mineral surfaces compared to cationic surfactants. Recent studies have also illustrated good potential for SEB using mixed surfactants, where the effectiveness can be enhanced at lower surfactant concentration. Zhu and Feng [74] found synergistic effects in PAH solubilization when mixtures of anionic and nonionic surfactants (SDS mixed with Triton X-100, Brij 35, and Triton X-305) were applied at a very low concentration due to formation of mixed micelles. The mixed micelles exhibited lower polarity and hence increased the molar solubilization ratio or micellar water partition coefficient at low CMC in the mixed surfactant solutions. Yu et al. [75] also demonstrated enhanced desorption and degradation of phenanthrene in SDS-Triton X-100 mixed surfactant solutions in soil-water systems. SDS reduced the sorption of Triton X-100 onto the soils. Mixed surfactants with a lower ratio of SDS promoted phenanthrene biodegradation while an increase in SDS in the mixed solutions had an adverse effect due to the preferential utilization of SDS by phenanthrene degraders.

### 3.2. Surfactant-Microorganism Interactions in SEB

**Figure 5:**
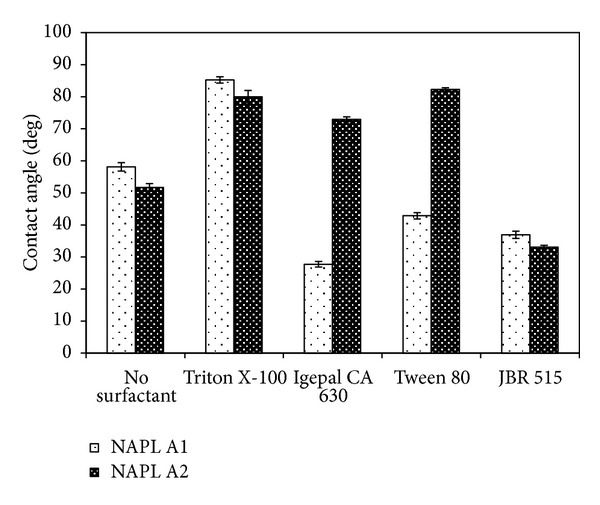
Water contact angles of *Burkholderia multivorans* (NG1) grown on model NAPL A1 and model NAPL A2 in the presence of surfactants. Prepared based on data published in Mohanty and Mukherji [11, 12].

It is imperative to conduct studies to decipher the surfactant- microorganism interaction which is unique in every scenario. The surfactant-microorganism interaction may directly impact the substrate uptake mechanism. As discussed in preceding sections, the direct uptake of hydrocarbons is favored by increase in cell surface hydrophobicity of the microorganism. Surfactants are reported to alter the cell surface hydrophobicity of microorganisms and affect the direct uptake of substrate from a NAPL or a solid phase.  Figure 5 depicts the change in the cell surface hydrophobicity of *Burkholderia multivorans* (NG1) in the presence of the three synthetic surfactants Triton X-100, Igepal CA-630, and Tween 80 and exogenously added biosurfacatant JBR-515 during the degradation of model NAPL A1 and model NAPL A2 [11, 12, 44]. The cell surface hydrophobicity of the naphthalene degrader, *Burkholderia multivorans* (NG1), was enhanced in the presence of Triton X-100 but not in the presence of the biosurfactant during the degradation of model NAPL A1 [12]. Triton X-100 not only caused an increase in surface area through emulsification, it also caused an increase in cell surface hydrophobicity and thus facilitated the degradation of aliphatic components in NAPL A1 through direct interfacial uptake (Figures 3 and 4). In contrast, the biosurfactant also caused an increase in degradation of the aliphatic components in NAPL A1; however, it employed the micellar solubilization mechanism in which hydrophobic cell surfaces are not a prerequisite for uptake. Emulsification coupled with increase in cell surface hydrophobicity was also reported to enhance the uptake of n-alkanes from diesel by *Burkholderia cepacia* [32]. NAPL composition also plays a significant role in the change in cell surface hydrophobicity (Figure 5). Increased cell surface hydrophobicity was observed during the degradation of model NAPL A2 rich in aliphatic hydrocarbons in the presence of all of the three chemical surfactants, whereas in case of model NAPL A1 (having much greater abundance of aromatic hydrocarbons compared to NAPL A2), increased hydrophobicity was observed only in the presence of Triton X-100 [12, 44]. 

**Figure 6:**
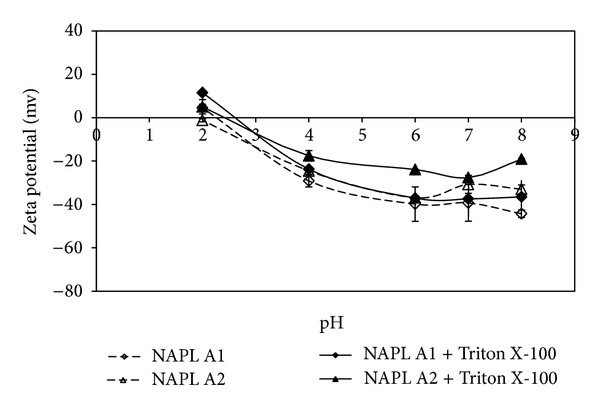
Zeta potential of *Burkholderia multivorans* (NG1) grown on model NAPL A1 and model NAPL A2 in the presence and absence of Triton X-100. Prepared based on data published in Mohanty and Mukherji [12].

The influence of surfactant-microorganism interaction is also manifested through changes in zeta potential which reflects the cell surface charge. Mohanty and Mukherji [12] observed that the zeta potential of the surfaces of *Burkholderia multivorans* (NG1) was less negative in the presence of surfactant Triton X-100 during the degradation of both NAPL A1 and NAPL A2 (Figure 6). The decrease in negative charge would favor attachment to the negatively charged NAPL droplets due to the weakening of mutual repulsion. Hua et al. [76] reported a similar observation for the biosurfactant (BS-UC) produced from *Candida antarctica* during the degradation of n-alkanes. Such changes are expected to affect the direct uptake mechanism. The change in cell surface charge and cell surface hydrophobicity may favor the adherence of the microorganism to NAPLs and to solid surfaces [77]. In a study by Kaczorek and Olszanowski [10], the progress of diesel biodegradation by *Pseudomonas alcaligenes* S22 over 21 days in the presence of surfactants was found to be related to the %adherence observed in BATH assay. Continuous degradation over 21 days was observed with saponin where the culture demonstrated high adherence in BATH assay over the entire period. In contrast, in the presence of Triton X-100, adherence in BATH assay was found to drop after 14 days and this hindered further diesel degradation. Alkyl polyglucoside (APG) surfactants, Lutensol GD 70, and Glucopon 215, derived from natural and renewable sources, have been reported to facilitate the biodegradation of diesel oil in *Achromobacter denitrificans* by facilitating greater adherence through cell surface changes as measured in the BATH assay [13]. Enhancement in diesel degradation was observed although the culture could degrade these surfactants. In contrast, preferential degradation of the APG surfactants hindered diesel degradation by *Stenotrophomonas maltophilia*. In another study by Rodrigues et al. [62], Tween 20, Tergitol NP-10, SDS, and CTAB applied at low concentration were found to interact with the cell surfaces of a PAH degrading *Pseudomonas putida* so as to alter the cell surface charge and the tendency to adhere to n-hexadecane in BATH assay. SDS and CTAB both lowered the cell surface charge significantly such that agglomeration of cells was observed, whereas Tween 20 enhanced the cell surface charge. None of the surfactants caused adherence of the cells to n-hexadecane water interface in the BATH assay. However, most of the surfactants enhanced the degradation of PAHs possibly by inducing cell surface hydrophobicity. It may be emphasized that the %adherence measured in the BATH assay is not a true measure of hydrophobicity, since this assay is strongly affected by the solution phase interactions and the choice of NAPL used in the BATH assay [32, 77]. Adherence to n-hexadecane does not reflect the ability of cells to adhere to PAHs. 

Some studies have shown that cultures with hydrophobic cell surfaces that typically tend to adhere to oil/hydrocarbons are adversely affected by the addition of surfactants [17, 78]. Their surfaces are altered after surfactant addition such that they are unable to adhere to oil/hydrocarbons and overall degradation is found to decrease. In contrast, cultures with hydrophilic cell surfaces may be benefited in the presence of surfactants as they may have greater accessibility to oil/hydrocarbons solubilized within surfactant micelle. Interesting effects are observed when multiple cultures with different uptake mechanisms coexist. Van Hamme and Ward [17] observed that in a coculture of *Rhodococcus* sp. F9-D79 and *Pseudomonas *sp. JA5-B45, degrading crude oil by direct uptake mechanism and uptake after solubilization, respectively, the *Rhodococcus* sp. primarily contributed to crude oil degradation in the absence of surfactants. In contrast, in presence of the surfactant, Igepal CO-630,the *Pseudomonas *sp. played a greater role in the degradation of aromatics in crude oil as Igepal CO-630 hindered the attachment of *Rhodococcus* sp. to the oil water interface. The change in hydrophobicity/surface charge that occurs in the presence of surfactants may thus depend on the inherent cell surface characteristics of the bacteria and its uptake mechanism, nature of the surfactant, and the manner in which it predominantly sorbs, that is, as monomers or as hemimicelles. Such changes have a strong influence on substrate uptake and the success of SEB. 

### 3.3. Biodegradability of Surfactants and Its Impact on SEB

As previously discussed, the addition of surfactants to a contaminated site adds to pollution due to the surfactant itself. Thus, based on environmental sustainability considerations use of biodegradable surfactants for SEB may be preferable over those that are recalcitrant and persistent. However, the solubilization capacity and effects of surfactants on the biodegradation of hydrocarbons need to be a key consideration governing their choice. Surfactant biodegradation is associated with various positive and negative implications. As discussed in the preceding sections, surfactants facilitate the utilization of petroleum hydrocarbons at higher rates by enhancing their bioavailability. Thus, degradation of the surfactants may hinder the uptake of petroleum hydrocarbons. In terms of their biodegradability and toxicity, surfactants may be classified as follows: readily degradable (either preferentially or nonpreferentially); hardly degradable, yet not inhibiting the degradation of other carbon sources; and posing toxicity to the microorganism and inhibiting its growth. The various key factors that influence the degradability are surfactant properties, degradation capability of the microorganisms, and environmental conditions. Surfactant properties include the charge (cationic, anionic, or nonionic), structural complexity (simple chain or with polymeric structures), and source (chemically synthesized or produced by microorganisms) [79]. 

The biodegradation of surfactants results from the ability of the microorganisms to catabolically assimilate the surfactant as carbon and energy source. As the structural stability of the micelles is destroyed, the hydrocarbon present in the micellar core may be released. Lee et al. [80] reported utilization of the Tween series of surfactants as primary growth substrate by PAH-degrading soil bacteria. Surfactant degradation occurred preferentially, and the fatty acid hydrophobic chain was selectively degraded, thereby making the surfactant more hydrophilic. Partitioning characteristics and HPLC chromatographic analysis revealed remarkable reduction in its surface-active nature and micellar solubilization capabilities, as reflected through increase in CMC and decrease in MSR. The utilization of the surfactant as carbon source may also have deleterious effect on hydrocarbon degradation due to the depletion of mineral nutrients/oxygen and the formation of toxic intermediates due to partial biotransformation of the surfactant. Preferential degradation of the surfactant itself may reduce the rate of contaminant degradation through a repression mechanism. In studies with Triton X-100, Wyrwas et al. [81] demonstrated the preferential utilization of Triton X-100 by a microbial consortium under aerobic conditions which consequently hindered diesel biodegradation. 

Positive effects of surfactant degradability include the removal of the surfactants from the polluted site and enhancement in the uptake of hydrocarbons. Sometimes, degradable surfactants serve as a primary substrate while the pollutant is degraded cometabolically [82]. In yet other scenarios where microbial cultures are capable of degrading both the surfactant and the hydrocarbons, culture growth on the surfactant may enhance the biodegradation rate of hydrocarbons. In studies by Bautista et al. [48], the biodegradable surfactant Tween 80 supported greater culture growth and yielded the highest biodegradation rate of naphthalene, phenanthrene, and anthracene by various bacterial cultures in comparison to other non-biodegradable and non-toxic surfactants. González et al. [67] reported similar results during the degradation of PAHs from contaminated soil by a bacterial consortium, where the biodegradable surfactant Tween-80 resulted in more effective PAH degradation compared to the non-biodegradable surfactant, Tergitol NP-10. 

Sometimes, bioavailability of the primary substrate can be improved with biodegradation of the surfactant due to greater release of hydrocarbons from the micellar phase into the aqueous phase, making the substrates more readily available to microorganisms. The release of hydrocarbons from the micellar phase is often found to be a limiting factor when nonbiodegradable surfactants are used for SEB. A biodegradable surfactant was found to stimulate the direct uptake of n-decane and n-tetradecane along with the uptake of micelle solubilized hydrocarbons [5]. Kim and Weber [14] reported the preferential utilisation and partial biodegradation of the Tween series of surfactants by a PAH degrading strain of *Sphingomonas paucimobilis*, which was unable to utilize the phenanthrene solubilized within surfactant micelles. Destabilization of the micelles caused release of phenanthrene into the aqueous phase which could subsequently be utilized by the culture. 

Although surfactant biodegradation has been reported, they are often only partially transformed and these transformation products tend to accumulate in the environment. Chemical surfactants are more resistant to degradation compared to biosurfactants that are readily biodegradable [83]. Numerous studies have attempted to determine the transformation products of ionic and nonionic chemical surfactants. The environmental condition is expected to have a strong influence on surfactant degradation. The degradability of cationic surfactants, which are highly biologically available owing to their charge, varies according to their type and the microorganisms involved. Studies have shown that anionic surfactant, LAS, and SDS are readily degradable under aerobic conditions at environmentally relevant concentrations [84, 85], although their degradation is less under anaerobic conditions. The degradability of nonionic surfactants varies with their structural complexity. Degradability of linear alcohol ethoxylates and fatty acid esters has been reported under both aerobic and anaerobic conditions; however, degradability is related to the number of ethoxy groups and alkyl chain length [15] and the specificity of the microorganisms [16]. In a study by Li and Chen [47], the biodegradability of nonionic surfactants was found to decrease with increase in chain length of the hydrophilic moiety of the surfactants such that degradability followed the order: Tergitol 15-S-7 > Tergitol 15-S-9 > Tergitol 15-S-12. Alkylphenol ethoxylates (APEOs), such as nonylphenol ethoxylates (NPEOs) and octylphenol ethoxylates (OPEOs), are only partially degraded in the anaerobic environment to form alkylphenols, such as, nonylphenol (NP), octylphenol (OP), and the corresponding monoethoxylates and diethoxylates. These transformation products are highly persistent in the anaerobic environment. 

Zeng et al. [79] compared the degradability of different types of surfactants (CTAB, Triton X-100, SDS, and rhamnolipids) along with glucose, an easily degradable carbon source, by *Pseudomonas aeruginosa*, *Bacillus subtilis,* and a microbial consortium obtained from municipal solid waste compost. It was observed that the cationic surfactant CTAB was toxic to the microorganisms, so neither glucose nor the surfactant was degraded. Triton X-100 was nontoxic; however, it was not degraded due to its structural complexity. The anionic surfactant SDS was easily degraded by all three microbial strains. The degradation of SDS is also reported in various other studies [86]. The biosurfactant, rhamnolipid was easily degraded by the consortium and the *Bacillus subtilis* culture but not by the *Pseudomonas* strain producing it. The compost microorganisms showed much higher degradation efficiency due to the diversity of microbial species compared to *Bacillus *sp., which showed low degradation efficiency and a long lag phase.

Mohan et al. [87] conducted biodegradability studies under aerobic, anaerobic, nitrate reducing, and sulphate reducing conditions. They analysed COD removal and gas production as the indicator for complete degradation using various microorganisms such as a nitrate and a sulphate reducer and *Vibrio cyclotrophicus *sp. Under aerobic condition, rhamnolipid and Triton X-100 depicted soluble COD removal efficiency of 74% and 47.1%, respectively, after 10 days. Under anaerobic, sulphate reducing and nitrate reducing conditions, COD removal efficiencies after 6 days were 47.2, 34.2, and 24.6% for rhamnolipids. The degradation of Triton X-100 was inhibited under these conditions. 

### 3.4. Toxicity of Surfactants

Surfactant toxicity is an important aspect which may adversely affect the SEB of oil and petroleum hydrocarbons. Surfactants applied at high concentration are often found to adversely affect the microbial growth on rich media or in the presence of easily degradable substrate. Surfactants added to resting cells at a high concentration have also been reported to cause a decrease in oxygen uptake rate. The concentration at which these adverse effects are manifested depends on the structure of the surfactant used and the nature of the microorganism. In contrast, growth inhibition and reduced substrate and oxygen uptake rate in the presence of oil and petroleum hydrocarbons are not necessarily a manifestation of toxicity since they may result from reduced bioavailability of the substrate. The toxicity of any surfactant is related to its capacity to adsorb and penetrate through the bacterial cell membrane [88]. Surfactant toxicity is caused by either of the two mechanisms, that is, disruption of the cell membrane by interaction with membrane lipids and by interaction of surfactants with protein molecules essential for cell functioning [5]. In addition to the surfactant type and dose, the toxic effect is also dependent on environmental conditions, such as solution pH. Cationic surfactants are more toxic at higher pH conditions (>7), whereas anionic surfactants are more toxic at lower pH conditions [14]. Surfactant toxicity to microorganisms has been studied using microorganisms capable of degrading oil and petroleum hydrocarbons in SEB scenarios and also using the bioluminescent organism, *Vibrio fischeri*. The latter studies have attempted to quantify the acute toxicity of surfactants based on effective concentration causing 50% inhibition in light output (EC_50_). This test provides a measure of nonspecific toxicity. Toxicity is found to increase with the increase in hydrophobicity which is commonly indicated by the octanol water partition coefficient, *K*_ow_ [89]. However, for surfactants, other measures of hydrophobicity provide better correlation. 

The ionic/nonionic nature of the hydrophilic head group on a surfactant affects its toxicity. Typically, nonionic surfactants are found to be less toxic to bacteria than ionic surfactants [46, 48, 73, 90–92]. Cationic surfactants, such as CTAB and TDTMA, exhibit greater toxicity than anionic surfactants, for example, LAS and SDS, which in turn exhibit greater toxicity compared to nonionic surfactants. Thus, primarily nonionic surfactants have been used in SEB applications. The Tween series of surfactants are least toxic [47] while Triton X-100 is reported to exert toxicity at higher concentrations [46]. 

In studies with branched and linear ethoxylated alkylphenols (such as NPEOs and OPEOs), Hall et al. [93] reported that the ethylene oxide (EO) chain length determines the toxicity of surfactants while the base structure of the surfactants (i.e., aromatic or aliphatic, branched or linear) does not have much effect on toxicity. The aromatic and aliphatic surfactants with EO molar ratios of 30 or higher were relatively nontoxic. Pavlić et al. [94] reported similar findings in algal toxicity tests using various species of algae. 

For nonionic surfactants with the same head group and similar structure, the alkyl chain length of the hydrophobic moiety is reported to affect toxicity. For alkylpolyglucosides (APG), the surfactant having the longest alkyl chain length was found to exhibit the highest toxicity [95–97]. In contrast, for structurally similar nonionic surfactants differing in the hydrophilic polyoxyethylene (POE) chain length, the toxicity was found to decrease as the POE chain length increased. Thus, the toxicity of polyoxyethylene sorbitan monooleates decreased in the order Brij30, 10LE, and Brij35 having 4, 10, and 23 POE units, respectively [73]. 

Surfactants with similar chemical structure varying in terms of their HLB value are reported to differ in terms of their toxicity to bacteria. It may be noted that HLB may increase due to the increase in hydrophilic chain length or due to the decrease in the hydrophobic chain length. The increase in HLB is thus expected to reduce nonspecific toxicity due to the reduction in hydrophobicity which inhibits its entrance into the lipid bilayer of the cell membrane [47, 98]. During the biodegradation of acetate and glucose, Triton X-165 (HLB 15.8) was found to be less toxic to mixed microbial cultures than Triton X-100 (HLB 13.5) [99]. Studies with luminescent bacteria and various groups of nonionic surfactants, such as fatty alcohol ethoxylates (FAEs), nonylphenol polyethoxylates (NPEOs), and alkylpolyglucoside (APG), have also confirmed the relationship between toxicity, structural characteristics and physicochemical properties (CMC, HLB, and interfacial properties) of surfactants [97]. For the APGs, EC_50_ was found to be dependent on the hydrophobic alkyl chain length, HLB and CMC of the surfactant such that toxicity increased as the alkyl chain length increased and HLB decreased. The toxicity of APGs increased as the CMC decreased. For fatty-alcohol ethoxylates, increasing the alkyl chain length lowered EC_50_ and increased toxicity, whereas increasing ethoxylation increased the HLB and lowered the toxicity. 

Most of the relationships between toxicity and structural characteristics of the nonionic surfactants revealed through tests based on bacteria are also found to be valid for higher organisms. Uppgård et al. [100] confirmed these relationships for fatty alcohols and ethylene oxides (*K*_ow_, hydrophobic chain length, HLB, and CMC) using a freshwater shrimp and rotifer species. However, tests based on bacteria only reveal nonspecific toxicity. Specific toxicity of surfactants may be observed through toxicity tests based on organisms, such as algae, rotifers, fish, shrimp, and snails. Toxicity of surfactants, when tested on different aquatic organisms show variable LC_50_ values depending on the sensitivity of the test species [88, 94, 101]. Most of these studies have revealed that the anionic surfactants are less toxic than nonionic surfactants, indicating significant specific toxicity associated with nonionic surfactants. In addition, surfactants and their degradation products also exhibit chronic toxic effects, such as endocrine disrupting activity. Degradation products of alkylphenol ethoxylates, such as NP and OP, are reported to fall in the category of endocrine disrupting substances (EDS). NP and OP are capable of inducing the production of vitellogenin in male fish, a protein that is usually found only in sexually mature females under the influence of estrogens [102]. Nonylphenols are highly toxic to aquatic organisms and possess the ability to mimic natural hormones 17-*β*-estradiol by interacting with the estrogen receptor. These transformation products are quite recalcitrant and tend to accumulate in aquatic sediments where they exert toxic effects towards plants and animals. 

## 4. Consequences of SEB

The introduction of surfactants into oil contaminated soil and aquatic environments may add to pollution through the accumulation of petroleum hydrocarbon degradation intermediates and partial biotransformation products of the surfactants. This may pose a threat to aquatic and terrestrial plants and animals. These ecotoxicological implications need to be considered for successful application of SEB. 

Complete removal of oil rarely occurs. Some components in oil are only partially transformed such that accumulation of intermediates may occur. As surfactants enhance the bioavailability, some components that are not inherently degradable by microorganisms may get partially transformed. Such partial transformation products that tend to accumulate are often more toxic than the parent compound. The intermediates are often acidic in nature and tend to cause pH drop in the system [12]. This may lead to the loss of viability of microorganisms due to adverse environmental conditions. Moreover, as the application of surfactants enhances micellar solubilization, it promotes desorption and transport of the sparingly soluble components in oil that typically remain sequestered through sorption. The mobility of PAHs and their penetration through sand was found to be increased when Corexit 9500A surfactants were applied to oil contaminated sand in the Gulf of Mexico [103]. Anaerobic conditions in the deep subsurface zone hindered their biodegradation and enhanced their persistence. Such mobilization may pose a threat of groundwater contamination. 

The application of surfactants in oil contamination scenarios may selectively facilitate the growth of specific microorganisms which may eventually hinder oil degradation [81]. The addition of biodegradable surfactants may change the microbial community structure as some hydrocarbon degrading microorganisms may respond by preferentially utilizing the surfactants instead of the hydrocarbons as reported by González et al. [67] for a PAH-degrading consortium. They demonstrated that a non-biodegradable surfactant supported a higher microbial diversity, whereas in case of a biodegradable surfactant only a few dominant species were present. 

Since surfactants pose a secondary source of pollution, there is much concern over the fate of surfactants in the environment once the site is remediated with respect to petroleum hydrocarbons. If the surfactants are eventually completely degraded by naturally occurring microorganisms, the surfactant aided remediation technology may be considered more sustainable. The fate of surfactants in the environment is dependent on their chemical structure and is controlled by the sorption and biodegradation processes. Cationic and nonionic surfactants have much higher sorption on soil and sediment than anionic surfactants. Various factors such as physiochemical properties of the surfactants, surfactant concentration, the nature of the sediments, and environmental parameters influence the sorption of surfactants onto sediment/soil [102]. Surfactant concentration in the environment is typically below their CMC value such that the surfactants exist as monomers. 

Bioconcentration of the surfactants and their degraded products in organisms pose another environmental risk. Bioconcentration increases with increase in hydrophobicity of the surfactants. For the highly hydrophobic long chain LAS homologues, the bioconcentration factors (BCFs) in rainbow trouts and fathead minnows are found to be in the range of 1.4–372 L/kg and 6–990 L/kg, respectively. Bioconcentration is more evident in aquatic ecosystems rather than in terrestrial ecosystems. Alkylphenols, such as NP and OP, which are common degradation products of alkylphenol ethoxylates, also tend to bioaccumulate in the aquatic environment due to their hydrophobic nature. Due to bioaccumulation, chronic toxic effects are eventually manifested in various organisms. Chronic toxicity of surfactants may be manifested at concentrations greater than 0.1 mg/L in the aquatic environment [104]. 

## 5. Conclusions

Based on the above observations reported in the literature, it is evident that surfactant-enhanced degradation of oil is a complex process. The success/failure of SEB depends on numerous factors including the choice of surfactant and its dose to be applied at the contaminated site, the hydrocarbon degrading microorganisms present in the environment and their response to oil/hydrocarbons, the interaction of the hydrocarbon degraders with the surfactants, and surfactant biodegradability and toxicity considerations. The structure of the surfactant, its HLB value, and its dose will affect the distribution of oil in the system through emulsification or micellar solubilization. The surfactant dose selected is expected to play a pivotal role in the success of the whole process. The administration of high dose may have a detrimental effect on hydrocarbon mineralization due to the toxicity of the surfactant to the hydrocarbon degrading microorganisms or due to the reduced bioavailability of the hydrocarbons solubilized within surfactant micelles. In spite of micellar solubilization and emulsification, the biodegradation of oil may be adversely affected if the microbial cell surface changes in the presence of surfactants are unfavorable. Although the choice of a biodegradable surfactant is not necessarily beneficial for petroleum hydrocarbon degradation, a surfactant that is eventually completely biodegraded will cause less ecotoxicological concerns. A surfactant that degrades only partially may not only have adverse effect on petroleum hydrocarbon degradation due to reduction in surface activity, but may also cause accumulation of recalcitrant intermediates in the environment. The adverse ecotoxicological implications of such a choice may make the surfactant aided biodegradation unsustainable. Moreover, the application of surfactants should be economically affordable. The successful implementation of SEB is thus very challenging and depends on all of the above parameters discussed. Due to the multiple challenges associated with this technology, field trials are strongly recommended before the direct application of the process on a large scale.

## Figures and Tables

**Table 1:** Studies on surfactant aided bioremediation of petroleum hydrocarbons, PAHs, oil, and model NAPLs by various microbial cultures.

Surfactant	Surfactant type	Substrate	Cultures	Reference
Hexadecyl trimethyl ammonium bromide (CTAB)	Cationic	Phenanthrene	*In situ* mixed culture	Chang et al. [49]

Sodium Dodecyl Sulphate (SDS)	Anionic	Diesel	Cold-adapted microorganisms	Margesin and Schinner [50]

Tween 80, Rhamnolipid (JBR)	Nonionic	Fluoranthene	*Pseudomonas alcaligenes *PA-10	Hickey et al. [51]

Triton X-100	Nonionic	Diesel	*Burkholderia cepacia* (ES1)	Mohanty and Mukherji [8]

Igepal CO-630	Nonionic	Crude oil	Mixed culture	Van Hamme and Ward [52]

Span 80, Corexit 9527	Nonionic	Crude oil	*Acinetobacter calcoaceticus* ATCC 31012	Bruheim et al. [53]

Triton X-100	Nonionic	Naphthalene	*Pseudomonas* sp.	Mulder et al. [54]

Triton X-100,Biosurfactants	Nonionic	Crude oil	*Bacillus* sp. B-UM	Wong et al. [55]

Biosoft EN 600, Igepal CO-630	Nonionic	Crude oil	Mixed culture	Ward et al. [18]

Biosurfactants, SDS	Anionic	Crude oil	Mixed culture	Urum and Pekdemir [56]

Crude Biosurfactant, SDS, Tween 80	Anionic, Nonionic	Aromatic and paraffinic hydrocarbons	*Pseudomonas* sp.	Anna et al. [57]

SDS	Anionic	Petroleum hydrocarbons	—	Khalladi et al. [58]

Tween 80 Triton X-100 Tergitol NP-10	Nonionic	PAHs (Naphthalene, Phenanthrene, Anthracene)	*Enterobacter, Pseudomonas, Stenotrophomonas *	Bautista et al. [48]

AT-7, Tween 80, L-10, Lutensol GD 70	Nonionic	Dodecane : Hexadecane (1 : 1)	Various strains of* Bacillaceae *and *Pseudomonadaceae *	Cybulski et al. [59]

Triton X-100	Nonionic	Phenanthrene		Jin et al. [60]

Saponin, Rhamnolipid, Triton X-100	Nonionic natural surfactant, Anionic glycolipid, Nonionic synthetic	Diesel oil	*Pseudomonas alcaligenes *	Kaczorek and Olszanowski [10], Kaczorek et al. [61]

Tween 80, Triton X-100 Surfactin, Rhamnolipids	Nonionic, Anionic biosurfactant	Total petroleum Hydrocarbons (TPH)		Lai et al. [37]

Tween 20 SDS CTAB	Nonionic Anionic Cationic	Fluoranthene Anthracene	*Pseudomonas putida * *ATCC 17514 *	Rodrigues et al. [62]

Tween 20, 80 Triton X-100 SDS	Nonionic Nonionic Anionic	Phenanthrene Pyrene	*Arthrobacter strain Sphe 3 *	Aryal and Liakopoulou-Kyriakides [46]

Brij 30, 35 Tween 80 Triton X-100	Nonionic	Pyrene, Phenanthrene, Naphthalene	*Pseudomonas putida *	Doong and Lei [63]

Tween 20, 40, 80 Triton X-100, Rhamnolipid	Nonionic biosurfactant	Pyrene	*Klebsiella oxoytca *	Zhang et al. [64]

Tergitol 15-S-X	Nonionic	Phenanthrene	*Neptunomonas napthovorans *	Li and Chen [45]

Brij 30	Nonionic	Naphthalene Phenanthrene	*Microbial consortia *	Kim et al. [65]

Tween 80	Nonionic	Pyrene	*Mycobacterium frederiksbergense *	Sarma and Pakshirajan [66]

Tergitol NP-10 Tween 80	Nonionic	Naphthalene Phenanthrene Anthracene	*Consortia C2PLO5 *	González et al. [67]

BS-UC, Mannosylerythritol lipids	Biosurfactants	*n*-Alkanes C8–C16 C11 : C14 : C16 (1 : 1 : 1)	*Candida antarctica *	Hua et al. [68]

Rhamnolipids	Biosurfactant	*n*-Alkanes in petroleum	Bacterial consortia	Rahman et al. [69]

Crude biosurfactant	Biosurfactant	TPH	Soil microcosms	Benincasa [70]

Rhamnolipids	Crude Biosurfactant	Oily sludge	*Pseudomonas aeruginosa, Rhodococcus sp. *	Singh and Cameotra [71]

Rhamnolipids	Crude biosurfactants	Crude oil	Soil microorganisms	Nikolopoulou and Kalogerakis [72]
